# The Clinical Advances of Oncolytic Viruses in Cancer Immunotherapy

**DOI:** 10.7759/cureus.40742

**Published:** 2023-06-21

**Authors:** Mohammed A Zolaly, Waleed Mahallawi, Zakaria Y Khawaji, Mohammed A Alahmadi

**Affiliations:** 1 Pediatric Hematology Oncology, Taibah University, Al-Madinah al-Munawwarah, SAU; 2 Medical Laboratory Technology Department, College of Applied Medical Sciences, Taibah University, Al-Madinah al-Munawwarah, SAU; 3 Medicine and Surgery, Taibah University, Al-Madinah al-Munawwarah, SAU

**Keywords:** herpes simplex viruses, adenoviruses, oncolytic viruses, virotherapy, cancer immunotherapy

## Abstract

A promising future for oncology treatment has been brought about by the emergence of a novel approach utilizing oncolytic viruses in cancer immunotherapy. Oncolytic viruses are viruses that have been exploited genetically to assault malignant cells and activate a robust immune response. Several techniques have been developed to endow viruses with an oncolytic activity through genetic engineering. For instance, redirection capsid modification, stimulation of anti-neoplastic immune response, and genetically arming viruses with cytokines such as IL-12. Oncolytic viral clinical outcomes are sought after, particularly in more advanced cancers. The effectiveness and safety profile of the oncolytic virus in clinical studies with or without the combination of standard treatment (chemotherapy, radiotherapy, or primary excision) has been assessed using response evaluation criteria in solid tumors (RECIST). This review will comprehensively outline the most recent clinical applications and provide the results from various phases of clinical trials in a variety of cancers in the latest published literature.

## Introduction and background

Cancer is defined as a group of disorders characterized by uncontrolled cell hyperplasia, abnormal division of cells, and the ability to invade other organs [1]. The ability to evade the host's immune system plays an essential role in malignancy formation. The primary way tumor cells establish a commendatory environment is by steering the immune response. This can be achieved through the expression of immunosuppressive molecules to inhibit the anti-tumor effects of immune cells; in particular, natural killer cells (NKs). programmed death-ligand 1 (PD-L1), indoleamine 2,3-dioxygenase (IDO), and Siglec-9 have been recognized as anti-apoptotic survival molecules in cancer [2].

The current conventional cancer therapeutic modalities include surgery, radiotherapy, chemotherapy, and targeted therapy. Noticeable progress has been achieved in the fields of gene therapy and immunotherapy. Virotherapy is a novel and promising approach to cancer therapy which is based on the competency of oncolytic viruses (OVs) to destroy malignant cells [3]. OVs refer to genetically modified viruses that selectively attack and lyse tumor cells but spare normal cells via different biological mechanisms [4]. Oncolytic properties of definite viruses were first observed accidentally in the 20th century when cancer patients who had viral infections experienced improvement and unexpected increases in their life span [5]. However, the lack of resources in genetic modification technology and the lack of knowledge in carcinogenesis were the most difficult barriers to generating a harmless virus that specifically invades and destroys neoplastic cells. However, recent evolutionary development in genetic engineering and viral genome modification has allowed us to manipulate viruses and direct them to specifically target malignant cells without producing any harmful effect on normal cells [5]. 

Our aim in this review was to address the most recent clinical applications and outcomes of OVs in cancer therapy and explore possible future advances.

## Review

Techniques and approaches that improve the applicability of OVs in cancer therapy

Redirecting Entry

The viral capsid modification approach was developed to facilitate the transduction and targeting of oncolytic viruses against tumor cells. This method is achieved by genetically inserting certain peptides into the viral capsid to contain specific ligands against receptors that are highly overexpressed by tumor cells, increasing the binding capacity and cancer entry of OVs [6]. Through this mechanism, herpes simplex virus (HSV) has been engineered to express folate-polyethylene glycol conjugate (FP-PEG), therefore obtaining the propensity to target folate receptor alpha, which is overexpressed in 40% of human cancers. Additionally, preclinical studies have shown increased efficiency of oncolytic HSV expressing single-chain fragment (scFv) antibodies against human epidermal growth factor receptor 2 (HER2) in redirection against HER2+ lung cancer cells [7]. 

Granulocyte-Macrophage Colony-Stimulating Factor (GM-CSF)

GM-CSF is a proinflammatory cytokine that has been widely utilized to promote the anti-tumor activity of OVs, and it has been identified to have a role in the regulation and stimulation of myeloid lineage cell proliferation and differentiation in bone marrow. GM-CSF induces tumor toxicity by promoting the maturation and function of antigen-presenting dendritic cells. As a result, the T-cell cytotoxic response against tumors is enhanced and potentiated [8]. Through this mechanism, GM-CSF has been proposed as an intriguing potential immunotherapy when combined with virotherapy. The first OV that was armed with GM-CSF was HSV-1. It was found to be an effective approach to ameliorating the lysis efficacy of HSV-1 in an animal lymphoma model. Since then, more OVs integrated and incorporated with GM-CSF have been developed [9].

Engineering OVs Armed With Cytokines

A further approach to developing OVs with highly potent anti-tumor effects is to arm OVs with multiple immunostimulatory and antineoplastic cytokines. For instance, IL-12, released by antigen-presenting cells (APCs), has many valuable features as an anti-cancerous cytokine. IL-12 stimulates the cell lysis activity of NKs and CD8 cytotoxic cells. Another anti-tumor effect of IL-12 that has been identified is its potential anti-angiogenetic ability, which may represent a powerful mechanism of anti-tumor activity. Therefore, many studies have reported that genetically engineered OVs with the IL-12 peptide inserted into their genome have remarkably inhibited tumor growth in animal models [10]. Examples of cytokine-armed OVs are given in Table 1.

**Table 1:** Examples of cytokines-armed OVs, mechanism of action, and cancer types in clinical trials MOA: mechanism of action, OV: oncolytic virus, IL-12: interleukin-12, NK: natural killer cells, HSV: herpes simplex virus, TNBC: triple-negative breast cancer, GM-CSF: granulocyte-macrophage colony-stimulating factor, DCs: dendritic cells, GIT: gastrointestinal, IFN-B: interferon beta, VSV: vesicular stomatitis virus, HCC: hepatocellular cell carcinoma, AML: acute myeloid leukemia.

Cytokines	MOA	Virus Family	OV variants	Cancer type
IL-12 [11]	NK and T cells activation. Antiangiogenic.	HSV	M032	Glioblastoma, astrocytoma, gliosarcoma
			ONCR-177	Solid tumors
		Vaccinia	TBio-6517	Colorectal cancer, TNBC
GM-CSF [12]	Activation of DCs	Adenovirus	ONCOS-102	Melanoma
		HSV	T-VEC	Melanoma, non-melanoma skin cancer, breast cancer, GIT tumors, ovarian cancer
		Paramyxovirus	MED15395	Solid tumors.
		Vaccinia	Pexa-vec	Renal cell carcinoma, HCC.
IFN-B [13]	Promote apoptosis, antiangiogenetic, enhance tumor antigenicity [12]	Vesicular stomatitis virus	VSV-IFNB+	HCC
			VSV-hIFNbeta-NIS [14]	T-cell lymphoma, AML, multiple myeloma.

Direct Immunostimulatory Effect

No less importantly, an additional mechanism that has been hypothesized is the direct apoptotic effects of OVs on tumor cells, which consequently activate the neglected immune response against these malignant cells. This activation can be justified by the direct killing of tumor cells by viral replication, which in turn induces apoptosis. The apoptotic malignant cells will release tumor-associated antigens (TAAs) in correlation with viral pathogen-associated molecular patterns (PAMPs) during therapy, allowing the infiltration of innate and adaptive immune cells within the tumor microenvironment. Because of this infiltration, leukocytes mobilize into the neoplasm, detect the cancer activity, and immediately eliminate it. Recently, the evidence pointed to the OV's ability to allow the anti-tumor immune response, which is known as the "kick-start" step for cancer therapy [15].

Delivery routes of OVs

The optimal OV route of administration is critical for achieving favorable treatment outcomes. OV should be perceived as a drug and meet the principles of pharmacokinetics to reach the site of mechanism and increase efficacy and bioavailability. If the administration approach was inadequate, the host’s innate and adaptive immune response would serve as a powerful limitation, interfering with viral infection and consequently abating the tumor lysis effects. Different delivery strategies have been applied for the sake of better success and response to treatment [16].

Direct intratumoral (IT) administration has received the most attention. The benefits of such a strategy include the ability to precisely control the concentration of OV inside the tumor and evade the undesirable systemic side effects of the infection. IT delivery is better suited to superficial tumors like melanoma, whereas glioblastoma may present operational difficulties in administration due to its deeper nature [17].

Intravenous (IV) administration of oncolytic viruses could be the most effective strategy for metastatic cancers. Unlike IT injection, IV guarantees higher systemic bioavailability to target any potential metastatic site. In order to be successfully delivered, patients must lack neutralizing antibodies against OV. Some viruses, however, could bypass the immune response even with the presence of antibodies [18]. Table 2 summarizes the benefits and ill effects of each route of administration [17].

**Table 2:** Routes of delivery of oncolytic viruses: advantages, disadvantages, and tumors in which routed OV: oncolytic virus, CNS: central nervous system, BBB: blood-brain barrier, LSCC: lung squamous cell carcinoma, GI: gastrointestinal

	Intertumoral route	Intravenous route	Intraperitoneal route	intrathecal route
Advantages	Higher concentration of OV in tumor site. Ability to control desirable concentration.	Good option in case of inaccessible tumors. Convenient and rapid.	Faster absorption. Relatively easy to be administrated. Targeting abdominal cavity organs.	Ideal for CNS tumors.
Disadvantages	Challenges in access deeper tumors. Difficult repeating doses in complex procedures.	Requires highly selective targets. Physiological barriers (e.g., BBB) and elimination by immune response. More toxicity.	Slower absorption than IV.	Limited to CNS.
Tumors	Melanoma Retinoblastoma Pancreatic carcinoma Astrocytoma Gliomas Breast cancer Colorectal cancer	Melanoma. Bladder cancer. LSCC Astrocytoma. Neuroblastoma. Ovarian cancer. Prostatic carcinoma. Glioblastoma	Angiosarcoma. Epithelioid sarcoma. Kaposi’s sarcoma. GI stromal cancer. Leiomyosarcoma. Liposarcoma. Pancreatic carcinoma	Glioblastoma. Glioma. Ependymoma. Primitive neuroectodermal tumor. CNS lymphoma

Limitations of OVs and how they can be overcome

The therapeutic outcome of OVs suffers from many defects that limit its effectiveness as a possible approach in cancer therapy. These flaws include, but are not limited to, the tumor microenvironment and the undesired immune response against OVs. 

One of the main issues is the restriction of spread and penetration through carcinoma. Extracellular matrix (ECM) and intracellular junctions act as physical barriers to the penetration of high molecular weight therapeutic agents [19]. In addition, the metaplastic property of malignant cells, which allows them to transform from mesenchymal to epithelial types, fastens the cellular junctions, creating an unperforated solid tumor and making intracellular penetration by OVs difficult [20]. In an attempt to solve this issue, preliminary induction of collagenase 108 or co-administration of hyaluronidase with oncolytic adenoviruses may lead to improved intracellular penetration of the virus, eventually enhanced therapeutic efficacy [21]. 

Another challenging area related to tumors is the hypoxia resulting from tumor growth and development. It has been shown that the hypoxic effect of tumors may interfere with the replication of OVs by preventing the progression of the cell cycle. As a result, OVs will be unable to undergo replication since they are cell cycle-dependent [22]. The innovation of modified OVs that conquer the hypoxic effects of the tumor has been studied, these studies even extended into more interesting modifications. Researchers have successfully customized adenoviruses with the ability of E1A genesis under hypoxic conditions. Thus, they take advantage of a hypoxic environment to make it a favorable condition for OVs [23].

Immunity can be a major obstacle in oncolytic virotherapy, as previous exposure or immunization can lead to a short lifespan. One possible approach to avoiding undesirable immune responses is to coat OVs with polymers in order to protect them during delivery, thus utilizing protected OVs with a longer half-life [20].

Finally, besides the above-mentioned limitations, there are other hurdles that may minimize the efficacy of virotherapy; these include viral tropism and disruption, delivery, and dosing strategies. However, unlike anti-viral immunity and tumor microenvironment, current strategies being constructed for previous defects have been tackled and improved due to vast enhancements in the field of virology and delivery platforms [6].

Viruses used as cancer immunotherapy

Several viruses have been tested for use as immunotherapies, such as adenoviruses, HSV, measles virus, and vaccinia virus. In the following section, we will discuss the most studied and investigated viruses.

Adenoviruses

Adenoviruses are non-enveloped viruses with double-stranded linear DNA genomes and an icosahedral capsid. The most promising and commonly used therapeutic adenovirus that has been heavily studied is the HAdV-C5 serotype [24-26].

Several genetic modification approaches have been recently developed to generate selective oncolytic adenoviruses with much less cytotoxicity [27]. The first approach was to induce small deletions in the vital adenoviral genes [28]. ONYX-015 was the first in the area of oncolytic adenoviruses, and it was further studied to evolve into a more potent adenovirus called Oncorine (H101) [29]. 

Herpes Viruses

HSV is a double-stranded DNA virus that has a large genome. The first oncolytic HSV was studied in the early 1990s. Either inactivation or deletion of the viral genome was used to inhibit viral replication in normal human cells. This genetic modification restricted virus replication and final cell lysis to actively replicate cancer cells. Two of the very early oncolytic HSVs that succeeded and progressed to clinical trials were HSV 1716 and G207. One of the most noticeable features of oncolytic HSV is its safety in comparison to other OVs. Thereby, HSV is a mighty candidate for genetic modification that would improve tumor selectivity and patient security [30,31].

Measles Virus

Measles viruses are RNA-genome viruses that are considered a type of the *Paramyxoviridea* family [32]. Measles virus could be genetically engineered to possess entry ligands for lysis cancer cells. The target that allows us to achieve the oncolytic efficacy of measles virus is the H protein, which can be found solely in measles virus tropism. [33].

Measles virus captured interest in the field of immunotherapy after a case of Hodgkin’s lymphoma remission following measles virus infection in 1949 [34]. The first oncolytic measles virus that was studied in a clinical trial is the Edmonston-Zagreb strain (MV-EZ). The adverse effects were mild, indicating high safety with the measles virus as an excellent OV candidate [35].

Poxviridae Viruses

Poxviruses are double-stranded DNA viruses known for their large size. One of the factors that makes poxviruses an exceptional candidate as OV is the fact that their replication cycle solely takes place in the cytoplasm, so they can’t attack normal human cells. Many viruses from the poxvirus family have been studied in the field of OVs. Following are the two most studied viruses from this family [36].

Vaccinia virus (VV): In 1977, VV was utilized by the World Health Organization as a vaccine for smallpox annihilation globally. Due to this, VV is considered safe for humans. Although VV naturally exhibits preferential growth rates in cancer cells, it can productively infect a wide variety of cell types, including non-dividing cells. Therefore, only genetically modified VV constructs selectively target tumor cells. Pexa-Vec (formerly JX-594) is the most studied type of VV and has completed dozens of clinical studies in multiple malignancies in more than 400 patients [37].

Myxoma virus (MYXV): MYXV is not pathogenic for humans, and it showed natural tropism for a wide spectrum of human malignancies. MYXV had been tested in preclinical studies in many animal models for different types of malignancies. The results of these studies showed the efficacy of MYXV as an OV. Even with the studies supporting MYXV safety and effectiveness, there are some points that should be addressed before initiating clinical trials such as approval of the delivery of the virus and good manufacturing practice (GMP) production of the virus [38].

Vesicular Stomatitis Virus

Vesicular stomatitis virus is a single-stranded RNA from *the Rhabdoviridae* family. It often spares human cells or can be asymptomatic, which made it an OV candidate. It is onco-selective due to type I interferon-dependent cellular immune responses, and it has a short replication time. Every year, new recombinant vesicular stomatitis viruses are created, being engineered to kill tumor cells [39].

New Castle Disease Virus (NDV)

NDV is a single-stranded RNA, non-segmented, enveloped virus known as avian paramyxovirus type 1 (APV-1) [40]. NDV infection in normal cells causes a strong response of IFN type 1. This immunostimulatory mechanism prevents cytotoxic effects and viral replication; thereby, it does not have the potential for infectivity in human cells [41]. The first clinical application of oncolytic NDV was performed on patients with acute leukemia in 1964. A year later, a clinical study on a patient with advanced cervix carcinoma was treated with IT NDV administration. Interestingly, NDV has been shown to induce tumor shedding and remission of lymph node metastasis [42].

Clinical experience with oncolytic viruses

Melanoma

Talimogene laherparepvec (T-VEC) is a modified HSV [43]. When T-VEC was examined in unresectable stage III-IV melanoma, the overall survival was 58% in the first year and 52% in the second year [44]. Subsequently, the outcome of the phase III clinical trial was an overall increase in survival rate, with outstanding results. Due to this, in 2015, T-VEC was approved for the treatment of unresectable stage III and IV melanoma by the Food and Drug Administration (FDA) [45]. 

A phase 1 study in uveal or cutaneous metastatic malignant melanoma showed that ICOVIR5 could extend to melanoma metastasis upon a single dose; however, it did not induce tumor regression. Systemic administration of ICOVIR5 might be superior for disseminated malignancies, as this trial concluded [46].

IT injection of PVSRIPO (recombinant nonpathogenic polio-rhinovirus chimera) with refractory unresectable melanoma. Four patients achieved an objective response (OR). A pathological complete response (pCR) was observed in two patients. The median follow-up time of 18 months showed no progression in six patients. The treatment showed a high safety profile with grade 1\2 adverse events [47]. 

Brain Malignancies

DNX-2401 is an oncolytic adenovirus that has been tested in recurrent malignant glioma. Eighteen patients were discerned to have favorable tumor reduction. The median overall survival (mOS) was 9.5 months, five patients had survived more than three years after treatment, and two patients had stable disease (SD). DNX-2401 was well tolerated with no toxicities [48]. 

In 2022, the results of a clinical trial of IT infusion of DNX-2401 in diffuse intrinsic pontine glioma (DIPG) were published. The MRI assessment at a median follow-up of 17.8 months, confirmed a reduction in tumor size. The response evaluation criteria in solid tumors (RECIST) showed partial response (PR) and SD during a median follow-up of 16.6 weeks. The mOS duration was 17.8 months, and two patients were alive roughly three years after treatment [49]. Furthermore, DNX-2401 was evaluated in patients with recurrent glioblastoma multiforme (GBM) and found to have an excellent safety profile. Tumor regression was observed in 20% of patients, and one patient with complete response (CR) was alive for eight years after treatment [50].

IT administration of G47∆, a HSV, in recurrent and progressive GBM was evaluated. Most of the adverse effects of G47∆ were limited to grades 1 and 2. The mOS was 7.3 months, and five patients achieved one-year survival, three of them survived > 46 months. SD and PD were observed in one patient each at two years [51]. A further study of residual or recurrent supratentorial GBM resulted in one-year survival. The mOS of all patients was 20.2 months after an initial dose of G47∆. The assessment of the safety of treatment was favorable, with the same adverse events as the previous study. The clinical benefit rate (PR+SD) was 100%. This study was the ground for the approval of G47∆ as the first OV in Japan [52].

Reolysin, a naturally occurring double-standard RNA virus, was utilized in recurrent glioma. The study reported well-tolerable treatment with no dose-limiting toxicities (DLTs), and prolonged survival rates among patients, with stabilization of disease in the majority of them [53]. A clinical trial of GBM patients who received reolysin. Most of the patients showed tolerable treatment with few side effects and an increase in mOS. These outcomes give a potential insight into combining ReoGlio with standard treatment in GBM [54].

AdV-tk, an adenovirus variant engineered to express the HSV thymidine kinase gene, was studied in pediatric brain tumors, including malignant glioma, anaplastic astrocytoma, and recurrent ependymoma. No DLTs were reported, and most of the adverse effects were grade 1 and 2. The two-year survival rate was 37.5%, the progression-free survival (PFS) and OS of the patient with ependymoma were greater than 47.7 months, and one patient with malignant glioma was greater than 37.3 months [55]. 

Head and Neck Squamous Cell Carcinoma (HNSCC)

A 72-year-old male with stage III laryngeal cancer was offered Rigvir, a naturally occurring ECHO-7 OV, as monotherapy. Rigvir was administered intramuscularly (IM) and subcutaneously (SC). A follow-up CT has shown no dissemination or local recurrence, a laryngoscopy demonstrated decreased tissue growth; and his initial symptoms have improved. Taking into consideration the solitary use of Rigvir, the patient was stabilized and preserved the functions of the larynx; this suggests that Rigvir could become a possible treatment for laryngeal cancer [56]. 

rAd-p53, GL-ONC1, and T-VEC were assessed in hypopharyngeal squamous cell carcinomas (HPSCC). The treatment showed auspicious results with tolerable, safe, and improved OR rates and disease-free survival rates [57-60].

Currently, NG-641 is being examined in surgically receptable HNSCC patients enrolled in an ongoing phase I trial. The trial is expected to be completed in August 2023 [61].

Lung and Pleural Cancer

TG4010 is a genetically engineered VV used in non-small cell lung cancer (NSCLC) stage IIIB/IV clinical trials performed in combination with standard treatment. All studies demonstrated an improvement in PFS and mOS rates. Regarding the safety assessment of treatment, adverse effects including flu-like symptoms, fatigue, anorexia, or neutropenia were observed [62]. 

A phase II clinical study published in 2017, aimed to determine the efficacy of reolysin in NSCLC. In conclusion, reolysin was tolerable with beneficial OR rates compared to standard chemotherapy treatment [63].

A 57-year-old female was diagnosed with moderately differentiated right lung adenocarcinoma and underwent radical resection. Two years later, she was admitted with a confirmed recurrence of lung adenocarcinoma. After the failure of her regimen and as a final hope, the patient was approved for experimental treatment with Oncorine. After the administration of four cycles of IT injection with Oncorine, the patient achieved SD with an improvement in performance score from 4 to 1 [64].

Currently, the following OVs are undergoing clinical trials in the management of lung cancer: RT-10 (NCT05205421), ADV/HSV-tk (NCT03004183), MEM-288 (NCT05076760), and YSCH-01(NCT05180851).

The efficacy of IT HSV1716 injection was evaluated in malignant pleural mesothelioma (MPM) not amenable to resection. The analysis of pleural fluid confirmed the replication of the virus in over 50% of patients. The treatment was generally well tolerated with no serious adverse effects; 50% of patients achieved SD status in eight weeks. This was the first clinical trial of using OVs in the management of MPM, and the results were justifiable for further clinical trials [65].

GI Cancers

Esophageal cancer: OBP-301, a type 5 adenovirus, was delivered by endoscopy in esophageal cancer. The adverse effect after initial treatment was transient self-limited lymphopenia, and the objective response rate was 91.7%. Local CR was observed with no malignant cells in the biopsy, including 83.3% in stage I and 60% in stages II/III, and PR [66].

Colorectal cancer: Enadenotucirev, an engineered adenovirus, was utilized in resectable colorectal cancer (CRC). An immune response was observed in 80% of patients with high local CD8 infiltration in the tumor. The delivery routes were both effective and tolerable. This phase 1 study supports the combination of Enadenotucirev with other immunotherapy modalities [67].

In another study, KRAS-mutated CRC patients received reolysin. The treatment was well tolerated. The clinical benefit rate (PR+SD) was 93.3%. The PFS and OS were 65.6 weeks and 25.1 months, respectively [68].

IT OH2 is an engineered HSV that has been investigated in esophageal and colorectal cancers. The virus injection was well tolerated, and adverse events of OH2 were mild in most patients. SD and PR were achieved in some patients, and tumor lesions were successfully regressed after treatment in four patients with lymph node and liver metastases. Those effects were identified as PR in the study and resulted from both [69].

Regarding clinical trials of OVs in CRC, there are numerous agents that showed efficacy and positive clinical outcomes, including E1-deleted Ad5, avipox virus, vaccinia fowl pox, JX-594, and NDV [70].

Pancreatic cancer: Unresectable locally advanced pancreatic cancer (LAPC) was treated with IT delivery of HF10, a genetically modified HSV. RECIST criteria implied three patients with PR, four patients with SD, and two with PD; the median PFS was 6.3 months, and the OS was 15.5 months. The overall clinical benefit (PD and SD) rate was 78%. Two PR patients had improved to achieve CR with resectable cancer and eventually underwent surgery [71].

IT injections of the oncolytic parvovirus variant (H-1PV) were administered in patients with resistant metastatic pancreatic ductal adenocarcinoma (PDAC). H-1PV was well tolerated with no toxicities. According to RECIST criteria and seven patients, one patient had confirmed PR and one patient had unconfirmed PR. The survival duration of both patients was 326 and 555 days, respectively [72].

Patients who were newly diagnosed with LAPC received an engineered adenovirus, Ad5-DS. Eight patients had SD, whereas one patient showed PR; this indicated a disease control rate of 100%, an OR rate of 11%, and a median PFS of 11.4 months. Regarding the safety profile, only grades 1 and 2 adverse events were observed [73].

Liver cancer: IT administration of JX-594 in patients with unresectable hepatocellular carcinoma (HCC) was tested. Transient flu-like symptoms were the main adverse effect. The study showed improved OS, and one patient had CR with tumor regression [74]. Another modified vaccinia virus is TG4023, which has been evaluated in liver tumors. Sixteen patients were assessed; eight of them had SD. The adverse events were mild, and the maximum tolerated dose was undetermined [75].

AdV-tk was administered to evaluate the postoperative recurrence of early-stage HCC. The OS rates in the first, third, and fifth years were 91.4%, 63.6%, and 52.1%, respectively, in patients who received AdV-tk. The gross examination of the tumor revealed a complete capsule and centric necrosis [76].

Gynecological Cancers

Breast cancer: Due to its tolerable safety profile, two clinical trials were conducted in 2021 to examine the effects of reolysin in early breast cancer and metastatic triple-negative breast cancer (TNBC). The preliminary data of early breast cancer studies demonstrated immunological infiltration of the tumor in more than half of the patients in the early stages of the trial [77,78].

IT delivery of AdV-tk was assessed in metastatic TNBC patients. CR was achieved in two patients (7.1%), one patient had PR (3.57%), and three patients had SD (10.7%). Thus, the overall clinical benefit rate was 21.43%. One patient who had CR remained disease-free without any systemic therapy for 39 months. AdV-tk was well tolerated and had an excellent safety profile [79].

Patients with stage II/III nonmetastatic TNBC were treated with T-VEC. Five patients (55%) attained pCR, which is equivalent to T0/N0, and three out of the five patients had previously tested positive for lymph node metastasis. The efficacy of T-VEC in refractory chemotherapy-resistant cancer could be manifested in one of the patients who had T0/N2 residual disease. Within the median follow-up of 27.9 months, no recurrence of the cancer was reported in all patients, and no DLTs were observed [80].

Ovarian cancer: GL-ONC1, a modified VV, was administered in conjunction with chemotherapy in two case reports of patients with recurrent, refractory stage III ovarian adenocarcinoma, and platinum-resistant ovarian cancer (PROC). It showed a significant improvement according to the level of CA-125 and CT pelvis findings [81]. The promising effects of these case reports indicate the superiority of combination therapy in cases of refractory ovarian cancer [82].

Olvi-Vec is a modified VV that was tested for PROC. The OR rate was 9%, the SD was 64%, the median PFS was 15.7 weeks, and three patients had an extended OS (33.6 months, 54 months, and 59 months). Olvi-Vec was tolerable and demonstrated promising clinical activity in patients with PROC [83].

The efficacy of enadenotucirev in recurrent PROC was discussed in a phase I trial. Initially, the agent was delivered via IP, but due to the catheter-related complication, it was replaced with an IV route. A total of 35% achieved SD, and 65% achieved a reduction in the targeted lesion burden. The combination of IV enadenotucirev and paclitaxel was well tolerated [84]. 

Urological Cancers

Prostate cancer: PROSTVAC-VF, a vaccine regimen directed against prostate-specific antigen (PSA), entered a clinical trial in patients with metastatic castration-resistant prostate cancer (mCRPC). The study showed significant improvement in mOS and PFS among patients who received the PROSTVAC-VF vaccine [85,86]. However, a phase III study in patients with mCRPC showed PROVSTAC-VF had no effect on OS as monotherapy [87]. Nevertheless, two recent clinical trials successfully demonstrated the potency of neoadjuvant PROSTVAC in driving T-cell infiltration inside the tumor microenvironment [88,89].

GEN0101 was evaluated in patients with CRPC delivered by IT and SC injections. There were no treatment-related serious adverse events observed. PSA did not decrease in all patients, yet its rise rates declined and were suppressed after the initiation of treatment. All patients who were assigned to the high-dose group achieved SD, and half of them showed a reduction in lymph node metastasis [90]. 

AdV PSA/MUC1/brachyury vaccination was injected in patients with mCRPC. The vaccine was well tolerated, with no DLTs reported. In respect of clinical efficacy, one patient had PR, five patients had SD greater than six months, and five patients showed a decline in PSA levels [91].

A current first-man phase I investigates the safety and anti-tumor efficacy of ORCA-010, a potency-enhanced replicating AdV, in treatment-naïve early-stage prostate cancer. An MRI of the low-dose group who had a significantly enlarged prostate confirmed a remarkable reduction in prostate size six months post treatment [92].

Bladder cancer: A trial of intravesical CG0070, an adenoviral vector, in patients with non-muscle invasive bladder cancer (NMIBC). The outcomes were a CR rate of approximately 50% and an excellent safety profile [93]. Thus, a phase II study assessing the safety and efficacy of CG0070 in bacillus Calmette-Guérin (BCG)-resistant NMIBC. The overall CR rate for the duration of 6 months was 47%, and 58% of them were patients with pure carcinoma in situ (CIS). The adverse events ranged from grade I to grade III [94]. The follow-up study revealed that the overall rate of CR in periods of six, 12, and 18 months was 44%, 30%, and 23%, respectively. A total of 35% of patients who were diagnosed with BCG refractory NIMBC had achieved CR in 18 months [95]. An ongoing single-arm phase II trial of CG0070 in unresponsive NIMBC to BCG. The preliminary results showed a CR rate of 87.5% at three months, with adverse events limited to grade I and II [96]. 

Nadofaragene firadenovec, a recombinant adenovirus, was employed in the treatment of NIMBC unresponsive to BCG. The treatment has a manageable safety profile. In patients who had CIS, 55 patients had CR for a duration of three months, and 25 patients remained CR for 12 months. With regard to high-grade Ta or T1 patients, 35 patients had a high-grade recurrence-free status for three months, and 21 sustained this status for 12 months. The two-year OS rates of patients with CIS and high-grade Ta/T1 were 91.1% and 93.5%, respectively [97]. 

Renal cell carcinoma (RCC): Pexa-Vec (JX-594) infusion was studied in patients with metastatic, refractory RCC. The treatment was well tolerated, one patient had achieved CR at week six, and the disease control rate was 76% [98]. A phase Ib study on patients with metastatic or unresectable RCC is currently being conducted. The initial data of the trial revealed a tumor burden reduction with an overall disease control rate of 75%: one CR, five PR, and six SD. The safety profile of the treatment regimen was acceptable, and grade 3 adverse events were mostly transient [99]. 

A case report of a 59-year-old male patient presented with chromophobe renal cell carcinoma stage 4 in the right kidney with histopathological confirmation of metastasis to both adrenal glands and liver and subpleural focus on the left lung. The patient underwent a right nephro-adrenalectomy. The treatment with IM injections of Rigvir alone was initiated. The follow-up CT imaging for more than one year, showed no disease progression, cancer metastasis, or lymph node involvement. Despite the estimated OS for patients with RCC stage 4, which equals less than 26 months, the patient survived over 33.7 months (remaining alive at the time of the report) and he never received any medical treatment except Rigvir [100].

Hematological Malignancies

Multiple myeloma: MV-NIS, an engineered measles virus, is in a phase I clinical trial in patients with relapsed and refractory multiple myeloma. Only one patient reached CR [101]. Reolysin was also tested in a phase 1 study in patients with relapsed multiple myeloma. The longest duration of SD time was eight months [102]. Regarding the safety profile, the maximum tolerated dose was not reached in both studies. 

Lymphoma: IT administration of TG1042, which is a modified adenovirus, was tested in a phase II clinical trial. Thirteen cases of primary cutaneous B-cell lymphomas (CBCL) were enrolled. Eleven patients exhibited an objective response; seven patients showed CR, and four showed PR. The treatment was tolerable, and the skin biopsy showed a good response to TG1042 [103]. 

A study of 15 cases of relapsed/ refractory hematological malignancies including seven patients with T-cell lymphoma (TCL) by using an IV administration of VSV. Three patients showed responses, PR for three and six months and a CR at 20 months. VSV-IFNβ-NIS as a single showed effectiveness in patients with TCL, with subsequent remission [104].

Leukaemia: A phase I study sought to address the clinical efficacy and tolerability of IT single dose of Ad-ISF35, a genetically altered adenovirus, among chronic lymphocytic leukemia (CLL) patients. The safety profile of Ad-ISF35 was regarded as acceptable and well-tolerated. Noteworthy, durable reductions in WBCs, lymphadenopathy, and splenomegaly were observed in a significant number of patients. Three patients had reached PR and seven patients had SD. Within a six-month period, six patients did not require additional therapies, and two of them had completed one-year treatment-free milestones [105].

A summary of clinical outcomes of various OVs in different neoplastic disorders is given in Table 3.

**Table 3:** Summary of clinical outcomes of various OVs in different neoplastic disorders mOS: median overall survival, ORR: objective response rate, CR: complete response, OR: objective response , DIPG: diffuse intrinsic pontine glioma, pCR: pathological complete response, PR: partial response, SD: stable disease, GBM: glioblastoma multiforma, MG: malignant glioma, AA: anaplastic astrocytoma, RE: recurrent ependymoma, AEs: adverse events, PFS: progression free survival, HNSCC: hand and neck squamous cell carcinoma, NSCLC: non-small cell lung carcinoma, MPM: malignant pleural mesothelioma, CRC: colorectal cancer, LAPC: local advanced pancreatic cancer, HCC: hepatocellular carcinoma, mBC: metastatic breast cancer, PROC: platinum resistant ovarian cancer, CRPC: castrate-resistent prostatic cancer, RCC: renal cell carcinoma, CBCL: cutaneous B cell lymphoma, CLL: chronic lymphocytic leukemia.

Cancer	Virus	Participants	Clinical outcomes	Reference
Melanoma	T-VEC	436	Improved longer-term efficacy and overall survival (mOS 23.3 months, ORR 31.5%, and CR 16.9%)	[45]
	PVSRIPO	12	OR: 33%, pCR in two patients, no progression in 50% in 18 months	[47]
	Poxvirus	1	Clinical regression of the lesion with grade ½ AEs	[106]
Pediatric DIPG	DNX-2401	12	mOS 17,8 months, Reduction in tumor size in 75%, PR: 25%, SD: 67%, 2 patients achieved 3 years-survival.	[49]
GBM	G47∆	19	mOS: 20.2 months, 1-year survival in 84.2%, PR: 1 patient, SD: 18 patients,	[52]
	Reolysin	15	mOS: 13.1 months, 2-year survival in 33%.	[54]
Pediatric MG, AA, and RE	AdV-tk	8	2-year survival: 37.5%, 2 patients > 36 months, AEs: Grade 1/2	[55]
HNSCC	GL-ONC1	19	PFS in 1^st^ year: 74.7% and in 2^nd^ year: 64.1% OS in 1^st^ year: 84.6%, 2^nd^ year: 69.2%	[57]
	T-VEC	36	ORR: 16.7%, six paitents had PR and 8 patients had SD.	[58, 59]
	rAd-p53	102	Improved OS and disease-free survival rates	[60]
NSCLC	TG4010	222	Improved PF survival among TG4010 compared to placebo, only 4% had grade 3/4 AEs .	[62]
	MEM-288	11	No DLTs. SD: 2 patients and PD: 5 patients.	[107]
MPM	HSV1716	12	SD: 50%	[65]
Esophageal cancer	OBP-301	13	ORR: 91.7%, CR: 61.5%, PR: 13%	[66]
CRC	Reolysin	30	PR: 20%, SD: 73.3%, mOS: 25.1 months	[67]
	OH2	54 (35 with CRC)	SD: 13 patients, PR: 2 patients, Tumor with lymph node metastasis regression in 2 patients	[69]
LAPC	HF10	9	PR: 33%, SD: 44%, mOS: 15.1 months	[71]
	Ad5-DS	9	SD: eight patients, PR: one patient, median PFS: 11.4 months.	[73]
	Reolysin	10	Disease control in 3 patients: 1 PR for 17.4 months, 2 SD for 9 and 4 months. Grade 1-2 AEs.	[108]
HCC	AdV-tk	77	Improved 5-year survival	[76]
	JX-594	30	Improved OS with CR in one patient	
mBC	AdV-tk	28	Clinical benefit: 21.43%, PR: 1 patient, SD: 3 patients, CR: 2 patients (1 patient remains disease-free for 39 months).	[79]
	T-VEC	9	pCR: 55% with lymph node metastasis regression in 3 patients,	[80]
	Reolysin	74	Improved mOS: 17.4 months	[109]
PROC	Olvi-Vec	12	SD: 64%, Improved OS in 3 patients > 30 months,	[83]
	enadenotucirev	38	SD: 35%, reduction in tumor burden in 65%	[84]
CRPC	GEN0101	9	SD: 67% and reduction of lymph node metastasis in 3 patients	[90]
	AdV PSA/MUC1/brachyury	18	SD: 5 patients, PR: 1 patient, Notable decline in PSA levels.	[91]
Bladder cancer	CG0070	35	CR in 6, 12, and 18 months: 44%, 30%, and 23% respectively	[94, 95]
	Nadofaragene firadenovec	157	Improved 2-year survival rate	[97]
RCC	Pexa-Vec	16	Tumor reduction and overall disease control 75%	[98]
CBCL	TG1042	11	CR: 63.6% and PR: 36.4%	[103]
CLL	Ad-ISF35	15	PR: 20%, SD: 46.7%, with reduction in lymphadenopathy and splenomegaly	[105]

The future of OVs in cancer therapy

**Figure 1:**
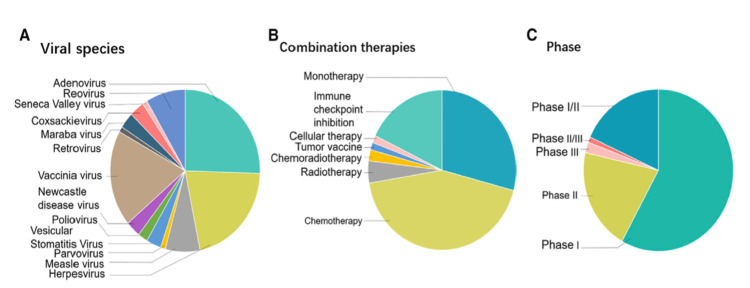
Overview of oncolytic virus clinical trials; 118 ongoing studies categorized on three bases: (A) Viral species, (B) Combination therapies, and (C) Clinical trial phase Image Source: Zheng et al., 2019 [6]. Use Permitted For non-commercial purposes: Creative Commons Attribution – NonCommercial – NoDerivs (CC BY-NC-ND 4.0)

The future implications of OVs have not been limited to previous examples. Clinical trials.gov details over 90 records on the use of potential redirected OVs that are currently conducted in clinical trials with a combination of therapeutic agents in different phases, with the initial promising outcomes in the regression of unresponsive cancers. However, less than 25 trials have proceeded to phase II/III [110]. Figure 1 gives an overview of OV clinical trials.

The combination of different cancer immunotherapy strategies such as chimeric antigen T cell therapy (CAR-T) and immune checkpoint inhibitors (ICIs) with OVs proved effective in tackling down tumor microenvironment [111]. OVs have the potential to promote the efficacy of cancer immunotherapy by altering the tumor microenvironment to make it more immunologically active [112]. CAR-T has revolutionized the arena of hematological malignancy management; however, its efficacy in solid tumors is still substandard. CARs are synthetic receptors composed of an extracellular antigen binding domain and one or more intracellular signaling domains that act in concert to activate the T cell upon antigen recognition [113]. In order to potentiate CAR-T therapy effects on solid tumors, a synergistic approach with oncolytic viruses was proposed. The facilitation of CAR-T cell therapy by OVs could be demonstrated in different mechanisms. The infection of malignant cells by OVs leads to a release of cytokines that recruit CAR-T cells. In further mechanism, the released chemokines and immunostimulatory cytokines have an augmentation effect on the function of CAR-T cell therapy, especially in solid tumor microenvironment [114]. Examples of the successful synergistic function of OVs with CAR-T cell therapy in animal models include the combination of CAR-T cell targeting folate receptor alpha combined with specific oncolytic adenovirus (OAd-BiTE) [115] and adenovirus Ad5Δ24 with CAR-T cell specific for tumor antigen GD2 [116]. Both studies showed a potentiating of cytotoxic efficacy, promoting CAR-T cell activation and proliferation with prolonged survival of mouse models. Combining ICIs and OVs is a method for overcoming the ineffectiveness of ICIs against cold tumors. The number of clinical trials investigating combinations of OVs and ICIs continues to rise, with the majority of available results demonstrating promising therapeutic potentials with excellent safety. The clinical translation of OVs and ICIs has the potential to upswing cancer treatment in the near future [112].

To sum up, the striking advances in the assimilation of virology, genetics, immunology, and carcinogenesis have a remarkable effect on developing directed OVs with good safety profiles and no observable toxicity or adverse effects. Future works are anticipated to be concentrated on establishing combinational strategies of optimized OVs with other anti-malignant therapies for cancer treatment [117]. Table 4 lists a selection of currently ongoing clinical trials that were carried out for various OVs with referral to ClinicalTrial.gov identifier.

**Table 4:** Selection of ongoing OVs in clinical trials (with or without co-therapy) obtained from Clinicaltrials.gov OV: oncolytic virus, P: phase, AdV: adenovirus, TNF: tumor necrosis factor, IL-12: interleukin 12, ICI: immune checkpoint inhibitor, PTD: protein transduction domain, NETs: neuroendocrine tumors, CD: cytosine deaminase, HSV: herpes simplex virus, TK: thymidine kinase, PC: pancreatic cancer, IFN: Interferon, GM-CSF: granulocyte-macrophage colony stimulating factor, FLT3LG: Fms-related tyrosine kinase 3 ligand, CCL4: chemokine ligand 4, CYP2B1: cytochrome p450 2B1, LTs: liver tumors, MV: measles virus, NIS: sodium iodide symporter, ATRT: atypical teratoid rhabdoid tumor, NAP: neutrophil activating protein, BC: breast cancer, GUCB: glucuronidase beta, B-gal: beta-galactosidase, GFP: green fluorescent protein, LC: lung cancer, NSCLC: non-small cell lung cancer, MM: multiple myeloma, BTs: brain tumors, GP: p-glycoprotein, CRC: colorectal cancer.

Viral species	Variant	Genetic modification	Condition	Co-therapy	P	Identification number
AdV	TILT-123	TNF-B and IL-2	Ovarian cancer	ICI	I	NCT05271318
	AdVince	Chromogranin A and PTD [118]	NETs	NA	I/IIa	NCT02749331
	Theragene	CD and HSV-1 TK [119]	PC	Radiotherapy	IIa	NCT04739046
	YSCH-01	L-IFN	Advanced solid tumors	NA	I	NCT05180851
	NG-641	FAP-TAc and CXCL9/CXCL10/IFN-alpha	Metastatic cancers	Chemotherapy or ICI	I	NCT04053283
HSV	G207	Deletion of γ134.5 [120]	Cerebellar tumors	Radiotherapy	I	NCT03911388
	OH2	GM-CSF	Advanced PC	NA	Ib/II	NCT04637698
	ONCR-177	IL-12, FLT3LG, CCL4 [121]	Advanced cutaneous and solid cancers	ICI	I	NCT04348916
	rRp450	CYP2B1 and Deletion of ICP6 [122]	Primary LTs	NA	I	NCT01071941
MV	MV-NIS	NIS	Recurrent medulloblastoma and ATRT	Surgery	I	NCT02962167
	MV-s-NAP	NAP	Metastatic BC	NA	I	NCT04521764
VV	GL-ONC1	GUCB, B-gal and GFP [123]	LC and mesothelioma	NA	I	NCT01766739
	MVA-MAGEA3	MAGE-A3	NSCLC	OV: ChAdOx-1-MAGEA3-NYESO, chemotherapy and ICI	I/II	NCT04908111
	JX-594	GM-CSF and B-gal [124]	Advanced BC and sarcoma	Chemotherapy	I/II	NCT026330368
Reovirus	Reolysin	NA	Relapsed or refractory MM	Chemotherapy	I	NCT02101944
			Relapsed or refractory BTs	Recombinant GM-CSF	I	NCT02444549
NDV	MEDI5395	GM-CSF [125]	Advanced solid tumors	Durvalumab	I	NCT03889275
VSV	VSV-hIFNbeta-NIS	IFN-beta and NIS	Stage IV or Recurrent Endometrial cancer	With or without chemotherapy	I	NCT03120624
	VSV-GP128	GP	Stage IV CRC	immunotherapy vaccines	I/II	NCT04046445

## Conclusions

Virotherapy has been shown to have a noticeable impact on malignant cells through direct toxic replicating effects and immunostimulatory activity at the molecular level, which leads to the induction of complex apoptotic pathways. Several viruses, including adenovirus, HSV, measles virus, and VV, have been manipulated and genetically engineered to possess an oncolytic effect. Depending on the desired site and stage of malignancy, OVs can be administered via a variety of routes. For instance, the IT route is used in melanoma, the IV route in disseminated cancer, and the intrathecal route in CNS tumors. Nonetheless, the tumor microenvironment and antiviral immunity might be potential pitfalls that restrict and minimize OV delivery and therapeutic effects.

Multiple clinical applications of OVs in combination with other standard or complementary treatment modalities have demonstrated outstanding objective clinical outcomes according to RECIST criteria in advanced late-stage cancers with tumor regression, prolongation of OS compared to standard treatment alone, and a manageable safety profile. Among different types of cancer, melanoma, brain tumors, CRC, and prostate cancer are examples of successful utilization of OVs in a clinical setting. Currently, there are a marked number of clinical trials evaluating the efficacy and safety of anti-neoplastic viruses in various types of solid tumors. We hope future successes will tackle the current challenges concerning the therapeutic limitations of virotherapy. 
